# Pedagogical strategies used in clinical medical education: an observational study

**DOI:** 10.1186/1472-6920-10-9

**Published:** 2010-01-28

**Authors:** Maria Skyvell Nilsson, Sandra Pennbrant, Ewa Pilhammar, Claes-Göran Wenestam

**Affiliations:** 1 University of Gothenburg, The Sahlgrenska Academy, Institute of Health and Care Sciences, Box 457, SE-405 30 Göteborg, Sweden; 2 Kristianstad University College, School of Teacher Education, SE-291, 291 88 Kristianstad, Sweden

## Abstract

**Background:**

Clinical teaching is a complex learning situation influenced by the learning content, the setting and the participants' actions and interactions. Few empirical studies have been conducted in order to explore how clinical supervision is carried out in authentic situations. In this study we explore how clinical teaching is carried out in a clinical environment with medical students.

**Methods:**

Following an ethnographic approach looking for meaning patterns, similarities and differences in how clinical teachers manage clinical teaching; non-participant observations and informal interviews were conducted during a four month period 2004-2005. The setting was at a teaching hospital in Sweden. The participants were clinical teachers and their 4th year medical students taking a course in surgery. The observations were guided by the aim of the study. Observational notes and notes from informal interviews were transcribed after each observation and all data material was analysed qualitatively.

**Results:**

Seven pedagogical strategies were found to be applied, namely: 1) Questions and answers, 2) Lecturing, 3) Piloting, 4) Prompting, 5) Supplementing, 6) Demonstrating, and 7) Intervening.

**Conclusions:**

This study contributes to previous research in describing a repertoire of pedagogical strategies used in clinical education. The findings showed that three superordinate qualitatively different ways of teaching could be identified that fit Ramsden's model. Each of these pedagogical strategies encompass different focus in teaching; either a focus on the teacher's knowledge and behaviour or the student's behaviour and understanding. We suggest that an increased awareness of the strategies in use will increase clinical teachers' teaching skills and the consequences they will have on the students' ability to learn. The pedagogical strategies need to be considered and scrutinized in further research in order to verify their impact on students' learning.

## Background

During supervised clinical training, medical students are expected to develop their professional competence and attitudes. The present study investigates how teaching is carried out during medical students' clinical training.

Often the literature declares that clinical medical education adheres to a master-apprenticeship system of learning and the fundamental condition for such teaching is that an expert is teaching a novice [1]. Consequently, in such a system of knowledge acquisition, the clinical teachers play a crucial role as a teacher. According to Lauvås and Handal the master-apprenticeship model focuses the students' ability to handle clinical praxis in accordance with what the clinical teachers believe is correct and what tradition allows [2]. Modelling [3] is central in the apprenticeship model and awareness of being a role model for younger colleagues and students in clinical practice is described among senior doctors [4,5] and deliberately used by clinical teachers [5].

The master-apprenticeship structure and modelling theory [3] are, however, not sufficient to meet modern academic educational demands. All formal education and academic teaching is aimed towards students gaining new knowledge and skills consistent with what is intended and necessary according to the curriculum. In medical education, as a consequence, everyday knowledge is expected to be left behind in exchange for scientifically-based knowledge or for knowledge based on professional experiential knowledge, useful in professional practice. Students' knowledge acquisition is, from this perspective, understood as a qualitative change from a previous kind of understanding. This means there are qualitative differences in how medical or clinical information is understood. Furthermore, such qualitatively different kinds of student understanding of subject matter may also be found among the students exposed to clinical teaching in a clinical situation. Consequently, we might expect qualitative differences in how something is understood among students. This stresses the need for clinical teachers to identify and take advantage of the students' qualitative differences in what they learn, understand and what they remember of what is studied [6].

Consequently, the way clinical teaching is carried out will have consequences on students' abilities to learn and understand. Ramsden describes three generic ways teachers can understand their role, each of which is related to how students are expected to learn [7]. Ramsden's three methods are: 1. teaching as telling or transmission of knowledge, 2. teaching as organizing student activity and, 3. teaching as making understanding possible. These three methods highlight important qualitative differences in how clinical teachers could consider teaching and student learning [7].

The effective and excellent clinical teacher is described as an: excellent role model; effective supervisor; and dynamic and supportive educator [8]. Kilminster and Jolly claimed that the essential aspects of clinical teaching are that it should ensure patient/client safety and promote professional development, and that clinical teaching has three main functions: educational; supportive; and managerial or administrative [9]. Kernan acknowledged that excellent clinical teaching is multifactorial, transcends ordinary teaching, and is characterized by teachers inspiring, supporting, actively involving and communicating with the student [10]. A number of studies emphasise communicative and supportive competence with the clinical teacher [8,11-14] and its importance for effective learning [15,16].

The literature demonstrates a vast number of pedagogical techniques used in clinical teaching [11,17-21], but there seems to be a lack of studies describing how such techniques are applied and used. In a review, Heidenreich stated that the majority of the teaching methods described were based on theoretical models and/or researcher's personal experience and not derived from empirical studies; and that the literature, to a large extent, is not focused on teaching performance but on the characteristics and behaviour of the effective clinical teacher [22].

One conclusion to be drawn from the literature is that clinical teaching must be seen as a complex learning situation influenced by the learning content, the setting and the actions and interactions of the participants. In order to increase the knowledge concerning clinical teaching, the aim of the present study is to explore how it is carried out in a clinical environment with medical students.

## Methods

### Design, setting and participants

Following an ethnographic approach rooted in symbolic interactionism, the focus was on people's actions and accounts in an everyday context and the aim was to look for meaning patterns, similarities and differences in how clinical teaching is carried out [23]. Non-participant observations and informal interviews were used as general data collection methods [23]. According to the ethnographic approach, the analysis of data involves interpretation of meanings, functions, and consequences of human actions and instructional practice, and how these are implicated in local, and perhaps also wider, contexts [23]. Consequently, in this study we used a qualitative design, with data collected from observations from clinical teaching situations and informal interviews with clinical teachers and medical students.

The data collection setting was a surgical ward at a teaching hospital in Sweden. Access to wards, clinical teachers and students was made possible by the Director of Studies at the Medical school and the department heads at the hospital.

In Sweden, the undergraduate medical education is extended over 11 semesters (5.5 years) before students graduate as doctors. The participants in this study were clinical teachers and their undergraduate medical students in their 8th semester taking a course in surgery. In order to focus on the interaction between the clinical teacher and the student, observation was mainly conducted during preparations and ward rounds (n = 21) at a unit where the physicians had time scheduled as assigned clinical teacher with students. With support from the clinical teacher, students were expected to manage patients. A few observations were also carried out at surgical outpatient clinics (n = 3), in operating rooms (n = 2) and during clinical lectures (n = 1). Data were collected on a total of 27 occasions (see table 1) during a four month period.

**Table 1:** List of observations, observer participated and settings.

Observation	Clinical teacher	Student	Setting
1	A	I	Surgical outpatient clinic, II*

2**	H	I	Surgical ward, I*

3	H	I	Surgical ward, I

4	C	II	Surgical ward, II

5	C	II	Surgical ward, II

6	C	II	Surgical ward, II

7	D	III	Surgical ward, III*

8	L	III	Surgical ward, III

9	K	III	Surgical ward, III

10	D	IV	Surgical ward, III

11	D	IV	Surgical ward, III

12	E	IV	Surgical ward, III

13**	E	V	Surgical ward, II

14**	E	V	Surgical ward, II

15**	E	V	Surgical ward, II

16	F	VI	Surgical ward, I

17	F	VI	Surgical ward, I

18	G	VI	OR, II

19**	H	VII	Surgical ward, I

20	I	VII	Surgical ward, I

21	I	VII	Surgical ward, I

22	B	VIII	Surgical ward, II

23	B	VIII	OR, II

24	J	VIII	Practical lecture, II

24**	E	IX	Surgical ward, II

26**	E	IX	Surgical ward, II

27**	E	IX	Surgical ward, II

* Hospital unit

** In this observation two observers participated.

Nine medical students (male = 2, female = 7; aged 24 to 37 years of age) were selected by the research group, and asked to participate. The students were selected to represent diverse ages, sexes and surgical units. Each student was observed on three different occasions where a total of twelve clinical teachers participated (male = 11, female = 1; aged 36 to 64 years of age) (see table 2).

**Table 2:** Teachers' age, title, clinical experience and experience as clinical teacher.

Code	Age	Title	Clinical experience	Years as clinical teacher
A	58	Specialist Consultant	32	23

B	58	Specialist Consultant	24	23

C	64	Specialist Consultant	30	*

D	65<	Specialist Consultant, Professor	40	*

E	42	Specialist Consultant, Med. Dr	18	7

F	42	Specialist Consultant, Med. Dr	18	6

G	36	Specialist Consultant	9	3

H	59	Specialist Consultant	30	30

I	50	Specialist Consultant	26	25

J	53	Specialist Consultant, Associate professor	26	10

K	55-65	Specialist Consultant	*	*

L	Ca 50	Specialist Consultant, Director of Studies	*	*

* The question was never asked or answered.

### Data collection

The observations were carried out by two researchers (MSN, SP). The researchers were both Registered Nurses (RN), PhD students in Health Care Pedagogics, and had extensive experience of work in health care. However, the presence of the researchers could affect the observations and this had to be considered [23]. In order to minimize the effect of the researchers they were adapted to the clinical environment by wearing a white coat with no nameplate which gave them access to the health care environment while at the same time showing they were not to be seen as health care personnel dealing with patients. In this way the researchers could participate without being a distraction or being directly involved.

The observations were guided by the aim of the study, during which the researchers took notes. Informal interviews were, for example, conducted in the coffee rooms or in the corridor. These informal interviews were mainly carried out in order to add further information to the observations and in order to establish a foundation for a deeper understanding of what had been observed. Observational notes and notes from informal interviews were transcribed after each observation.

### Ethical Considerations

Permission to carry out the study was given by the head of each department. Informed consent was obtained from students and clinical teachers in accordance with the Declaration of Helsinki [24] after they were informed of the purpose, method and publication of the study, that participation was voluntary, and that they could withdraw from the study at any time. When this study was planned and conducted, no approval by an ethics committee was required for this type of study according to Swedish law. In the observations where patients were present, the patients were informed of the purpose of the study and that the researchers were bound by professional secrecy in their role as health care personnel.

### Analysis

Data analysis was performed in two steps. Step I: A preliminary analysis was carried out when observational notes were taken. This analysis resulted in intuitive hypotheses, such as, is this pedagogical technique? Is this a way of showing the student something? These intuitive hypotheses were tested in relation to further data collection. The analysis process was in this way iterative and undertaken throughout the research process, allowing for intuitive hypothesis to be tested with further data collection [25]. In this first analysis step the researchers discovered that clinical teachers used different strategies in teaching students.

Step II. When all the data material was collected and transcribed, the data text was read several times and meaning units describing different ways of teaching detected. These meaning units were given a code describing their content. Data text, meaning units with entailed code were read several times and seven different pedagogical strategies could be described as a result of this final analysis. Furthermore, the results of the analysis were discussed in the research group (MSN, SP and EP) until agreement was reached (see example of the analysis process, table 3).

**Table 3:** Example of analysis process

Field notes	Meaning unit	Code	Pedagogical Strategies
**From observation during sitting rounds (pre-round conference)** The nurse starts the round run-through by informing that the patient is experiencing pain under the right fossa. The teacher turns to the student and asks: She has a long anamnesis, what could it be? The teacher and student discuss different conceivable diagnostic alternatives and possible investigations. The teacher concludes: This case has now become a case for investigation, a patient who should be in hospital but can be examined via the home.(13)	*The teacher turns to the student and asks and they discuss*.	*Questioning*	*Questions and answers*

**From observation during sitting rounds** The teacher *systematically goes through the medication the patient is taking and explains the effects of the medication*. (2).	*The teacher explains the effect of the medication*	*Lecturing*	*Lecturing*

**From observation of clinical teaching in the patient's room** The student leads the conversation with the patient. The teacher stands behind and listens. The atmosphere is calm and harmonious. Everyone (teachers, department doctors and three students) is standing round the patient watching while the student palpates the patient's abdomen. The teachers interject with questions to the patients: Can you cough? The patient: I avoid coughing. *The teacher takes over the conversation with the patient by asking more questions*. This transition between student and teacher feels smooth and natural. (1)	*The teacher takes over the conversation with the patient by asking more questions*.	*Supporting*	*Supplementing*

**From observation during sitting rounds (pre-round conference)**. The teacher tells the medical student that the patient has a typical pronounced "wide gait ataxic walk" The medical student doesn't know what that looks like. *The teacher illustrates this and explains how it is caused*. The medical student says; Aha that's what it looks like! (11)	*The teacher illustrates this*.	*Showing*	*Demostrating*

**From observation of clinical teaching in the patient's room** After the medical student and the teacher have sat and discussed the patients' status at sitting rounds, it's time to meet the patients. The medical student who is in charge of the patient, asks the patient; may I take a look at the wound? The patient says; yes, of course. (The medical student addresses both the patient and the teacher by looking at them both). *When she asks the question, the teacher answers the patient and takes over the consultation*. The medical student seems somewhat "left out". The medical student looks at the teacher who subsequently takes over the consultation completely. (3)	*When she asks the question, the teacher answers the patient and takes over the consultation*	*Taking over*	*Intervening*

## Result

The result of this study shows that the clinical teacher uses a number of pedagogical strategies in clinical teaching, in order to increase the likelihood of student learning. The strategies are entitled; 1) Questions and answers, 2) Lecturing, 3) Piloting, 4) Prompting, 5) Supplementing, 6) Demonstrating and 7) Intervening. These strategies are described below together with selected observational notes in order to support and clarify their meaning.

The clinical teacher frequently made use of these strategies to help the students solve problems or complete tasks. The strategies were used flexibly and could be changed during clinical teaching depending on situation, context and preferences of the clinical teacher.

### 1. Questions and answers

This strategy is observed when clinical teachers ask questions in order to activate the students; make them discuss and describe how to deal with medical problems; and management specific to the patients. The teachers' point of departure is the students' reasoning in combination with their own preferences in the main focus of the clinical problem. The teacher occasionally made a conclusion, summarizing the student's thoughts and argumentations.

A patient with kidney problems is discussed.

Teacher: On the x-ray, it's hard to tell the difference between pus and fluid. How do you figure out what it is?

The student picks up the sample test has a look and answers.

Teacher: He has low creatinine, why?

The teacher and student further discuss what the cause of the problem could be.

Teacher asks: What is it we want to know? What do we want an answer to? What do you want to know about the kidney's function? What do you look at then?

Student answers. The teacher nods and confirms. The teacher ends the discussion by saying that we may possibly talk to the urologist about this patient (I (clinical teacher, see table 2). 20 (observation, see table 1)).

The teacher also permitted the students to ask questions and relate these to the teachers' reasoning and actions. There were also examples where a student's question was returned by the teacher with the comment: *The problem and solution are now your responsibility*.

Using this strategy, the teacher created a dynamic process where the clinical teacher and students shared newly encountered experiences with previously acquired knowledge and experience.

The strategy sometimes took the form of an examination. For example, in one situation a teacher asked: *What is a hernia*? *How long will the patient be on sick leave? *The questions asked were based on what the teachers considered most important to understand. The teacher would supplement with knowledge they considered crucial, which could result in lecturing.

### 2. Lecturing

By asking questions and observing students' behaviour, the clinical teacher could assess students' level of knowledge. In cases where students showed a lack of knowledge, the teachers' intention changed from questioning to lecturing about the actual area of knowledge. Lecturing could also occur if teachers observed errors in any areas or a deficit in students' behaviour or reasoning. Lecturing took place frequently throughout the teaching session and examples of the strategy included: defining the meaning of medical terms; explaining symptoms of illnesses and localisations; and surgical and medical treatments. The clinical teacher clearly explained what areas of medical treatment required the most attention. Lecturing not only included medical theories and facts, but also, implicitly, medical attitudes and guiding principles in problem solving: for example, how to act and communicate with patients in consultation. The observational note below illustrates such a situation.

The clinical teacher clearly and precisely describes the procedure a doctor should go through when examining and talking to a patient who has an interpreter present. The teacher explains what to think about and how to conduct oneself with both the patient and the interpreter (A, 1).

### 3. Piloting

The meaning of this strategy is that the clinical teacher uses guiding questions, statements or signals to ensure the student pays attention to and focuses on specific content in order to reach an expected or previously decided goal. By piloting, the teachers prevent students from getting stuck in the management of a particular task. The teachers used guiding statements, invitations or questions in order to make them continue what they were doing. The students acted according to the teacher's directives, but the students' understanding and reasons for their actions were not discussed and there was no request for critical thinking or understanding from the teacher. Easing the student's actions by piloting does not necessarily lead to the intended perception or increase of knowledge. Students acted according to the teacher's directives without discussing the meaning or intended goal. In such situations there was no request for critical thinking or understanding from the teacher. Consequently, by piloting, the teachers guide the students around the difficulties in a clinical situation. The observational note below illustrates piloting when discussing postoperative management. In this situation the teacher directs the student in what to focus on, in order to get the postoperative management completed and done.

The medical student prescribes fluids. The nurse writes this down. The teacher nods consent.

The teacher says: We should take tests.

(Then the tests were prescribed by the teacher)

The teacher continues: What about an analgesic?

The student asks how much the patient should have (H, 19).

Piloting could also be used by the clinical teacher when they aimed to place students in a situation where they were expected to develop their understanding or/and experience-based knowledge. A situation which often occurred was that the teacher pointed out that students should meet and talk with the patients before making any judgments concerning treatments or assessments. In this situation, the teacher seldom specified or discussed what they wanted the student to learn or experience. Consequently, when piloting was used it was difficult to know whether the meaning was understood by the students.

### 4. Prompting

This strategy is characterized by the clinical teacher prompting a student to prevent the student "losing face" in front of the patient or other personnel. This approach is similar to piloting, but the focus of using prompting is found in the process. By prompting, the teacher supported the student in, for example, communication with a patient; whilst using piloting, the purpose was to direct the student to the correct answer or action. Accordingly, by prompting, the teacher supported the student in adopting the role of doctor. This approach was observed in situations where the students appeared to need help in their assessment, problem solving or in communication with patients or nurses. The teacher provided advice and/or directives by prompting. One illustration of this is described below.

The teacher is standing away from the bed. The medical student seems unsure if the wound appears to be healing and subsequently looks at the teacher. The teacher whispers to the student: The wound looks like it's healing fine.

The medical student then relays this to the patient (H, 3).

### 5. Supplementing

This approach is characterized by clinical teachers' supplementing during students' communications with patients or other personnel. The strategy is characterised by the teachers either adding some complementary important facts, or in some cases completely taking over the student's communication. This strategy demands teachers' sensitivity and awareness in deciding whether students are in need of support to handle a situation, otherwise loss of face is inevitable.

The student greets the patient. The student sits on a stool in front of the patient who sits on the bed. The teacher stands nearby and listens while the student talks to the patient. After a while the student signals (by looking at the teacher) that she has nothing further to say. The teacher then nods and brings the conversation to an end (A, 1).

In this particular case, the student signals that she does not know how to deal with the situation entirely. The teacher notices this and supports the student by helping her with what has to be said. In other cases the clinical teachers assessed the students' ability to deal with the situation and found it necessary to step in and supplement in order to continue the consultation, sometimes together with the student.

### 6. Demonstrating

With this strategy the clinical teacher demonstrates how to act, assess, communicate, and perceive a problem. This is demonstrated when teachers deliberately illustrate how to act or what to focus on, by displaying the correct behaviour in a clinical situation; for example when communicating with patients, or in assessment or evaluation. The observational note below describes such a situation.

Instead of the teacher telling the student what to ask the patient, the teacher does it himself and palpates the patient's abdomen, whilst the student observes (E, 14).

Demonstrating also included situations where the clinical teacher facilitated student perception of the learning object (seeing, hearing, listening or feeling). The purpose was to illustrate and create a perceptual understanding of a physical phenomenon. For example by evoking or pointing out medical phenomena or symptoms as described in the observational note below.

At ward rounds the teacher examines a patient with a fluid filled abdomen. The teacher says: Look here (the teacher then does a vibrating motion with his hand on the abdomen) do you see the wave motion in the abdomen? When it looks like this, there is a lot of fluid in the abdomen (F, 17).

The strategy also covered the clinical teacher taking the patient role, in order to clarify typical symptoms. Another example of demonstrating can be found in the operating room, where students were encouraged by the teacher to increase their awareness of the structure and abnormalities of an organ.

### 7. Intervening

Significant in this strategy is the teacher taking an authoritative role, interrupting the student and taking over the situation. In intervening, the clinical teacher focuses on getting the assignment completed. The observational notes below describe one situation where a teacher uses this strategy.

At ward rounds one of the medical students, who is in charge of the patient, asks; "may I look at the wound?", and the patient says, "yes of course." The medical student asks both the patient and the teacher by looking at the teacher. As a result, the teacher responds and takes over the consultation, leaving the medical student feeling somewhat "excluded". The medical student looks at the teacher who subsequently assumes complete control of the consultation (B, 3).

Significant in the above situation is the student's actions being interrupted when the clinical teacher intervenes and takes over. The student has to stand aside and assume the role of an observer. Using this strategy, patient management, organisational demands and limitations were demonstrated to the student. We observed that the students could thus experience a lack of feedback resulting in a lack of explanation and diminished understanding of their actions and how they managed the situation. Sometimes they felt "excluded" and their knowledge undervalued.

## Discussion and Conclusion

The aim of this study was to explore how clinical teaching was carried out in clinical education. The study was carried out in several clinical units at the largest teaching hospital in Sweden. By observing clinical teaching and interaction in authentic situations, a more comprehensive understanding of the educational mechanisms of clinical teaching could be reached. The result is mainly built on the observations made during clinical teaching, and the informal interviews were generally used to support the understanding of the observed phenomena. Research from other settings and levels in medical education, would be required to determine whether the pedagogical strategies described reflect a more general way of teaching medical students. More likely is that the pedagogical strategies are related to a number of factors such as students' knowledge level, clinical situation and personal preferences, but also to educational culture at the clinic. Therefore, other strategies might be observed in other settings or situations. We assume however, that the findings demonstrate the importance of attention to pedagogical strategies used in clinical teaching in order to facilitate student learning.

The findings of this study elucidate that clinical teachers used a repertoire of different pedagogical strategies namely: *Questions and answers, Lecturing, Piloting, Prompting, Supplementing, Demonstrating and Intervening*. Comparable behaviours in clinical teaching have previously been described in the literature [8,22] For example questioning [21,22] or dynamic teaching provides explanation, answers questions, giving directions and directing learning [8]. Even the supportive role as a supervisor that gave opportunities for the student to be involved in patient care have been described [8]. Almost all literature focuses on characteristics and behaviours of an effective clinical teacher and not on teaching methods [22]. Few empirical studies have been conducted in order to explore how clinical supervision is carried out in authentic situations. Therefore, this study adds to previous studies by giving empirical evidence of a teaching repertoire used by supervisors in clinical supervision. This result shows that clinical teaching is complex and diversified. In this study we have shown that clinical teaching consists of a spectrum of several teaching alternatives, and is not previously described in this way in the research literature. In accordance with the research literature, the descriptions of the pedagogical strategies also give evidence for clinical teachers' threefold function and role concerning: education; management; and support [9]. In this conclusion we focus on the educational aspect of the pedagogical strategies.

**Figure 1:**
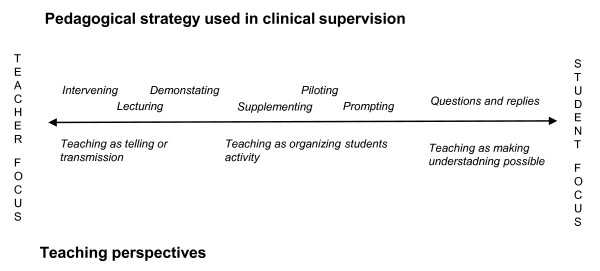
**The relationship between pedagogical strategies used by clinical teachers and the superordinate teaching perspective previously suggested by Ramsden (2003)**.

In accordance to Ramsden the pedagogical strategies, can be divided into different superordinate ways of understanding teaching and learning. The strategies bring into the open three underpinning ways of understanding how to teach namely: teaching as telling or transmission; teaching as organizing students' activity; and teaching as making understanding possible [7]. Each of these teaching perspectives has consequences on the teachers' focus in clinical teaching. Figure 1, illustrates the relation between pedagogical strategies, underpinning teaching perspective and the teachers' focus. The illustration should be seen as a continuum, where the pedagogical strategies placed to the left comprise a primary focus on teachers' own knowledge and acting; while the strategies described at the right end of this continuum comprise a focus on students' activity and understanding. These perspectives and focuses will be further discussed below.

The descriptions of the strategies *Lecturing, Demonstrating and Intervening *are in accordance with the teaching perspectives viewing teaching as *Telling or transmission of knowledge *[7]. This perspective is the most traditional and common perspective of teaching in higher education, where teaching is seen as a transmission of authoritatative content or the demonstrations of procedures [7]. The teacher is required to be an expert in the subject matter and could be seen as the store of undistorted information. The focus in teaching is on the teachers' personal knowledge and how it can be transmitted efficiently [7]. In applying the strategies, *Lecturing *and *Demonstration *the teacher focus was directed toward own acting and knowledge i.e how the subject matter could best be expressed. The students' understanding of the transmitted content was rarely discussed. This teaching perspective could also be linked to situations where it was not possible to allow the students to complete their actions, and the teacher had to *Intervene*. By *Intervening *the teacher demonstrated for example the way to act, or the organisational limitations. This could be seen as a transmission of knowledge about a clinical situation or organisational demands. However, by using this strategy, the action planned by the students was often interrupted. Consequently, this strategy could also impair the students' learning process which could result in a deterioration of the relationship between teacher and student.

By *Piloting*, *Prompting *and *Supplementing *the teacher supported the student in taking the role of an active doctor handling the clinical situation and guided the students on how to react. Consequently, these approaches were in accordance with a teaching perspective seeing *Teaching as organizing students' activity *[7]. In applying this teaching perspective, the focus is moved from the teacher toward the student's activity and the central role for the teacher is to help the student to be active. It is also assumed that by supporting the student in experiencing their acting, learning will take place [7]. For instance, even when the students make errors and experience the consequences of their actions, learning will occur. Learning by experience means that education, like life, is a process of continuously reconstructing experience. The starting point of the activity should be the learner's need for knowledge and the teacher's role is not to control the learning situation, but rather to act as a resource person guiding the situation [26]. It is also assumed that learning how to reflect on what we do and to apply our own knowledge to new situations follows naturally [7]. Improving teaching from this point of view is about extending lecturers' repertoire of techniques [7] i.e. to use the most appropriate strategy/techniques to support the student in acting.

The findings suggest that the clinical teachers' method of support is of great importance and it was essential that the teacher showed sensitivity and stepped in only in a supportive manner, even though their presence and intervention increased several students' confidence (should any potentially harmful mistakes be made with the patient). How important the teacher-pupil relationship is in clinical education is well established [8,12,15,27] not only where examples of effective relationships have proven to enhance learning, but also where examples of poor relationships have compromised a students learning [16].

Another characteristic in these findings was that students' understanding of the clinical situations and actions were rarely discussed or explored by the teachers and, notable during observations was that the individual students seemed to not always understand the consequences and motive of directions and actions. Discussions concerning learning objectives were rarely introduced or called attention to by the teacher. Neither was the content selected to adjust and facilitate understanding by the students. Consequently, the students were mostly left alone to figure out how the knowledge transmitted, demonstrated or experienced could be understood and made useful in other clinical situations. *Questions and answers *though, could be seen as a strategy that was aimed at *making understanding possible *[7]. By using this strategy, the focus turned to the students' own understanding of the clinical situation. According to Ramsden teaching should be comprehended as a process of working cooperatively with learners to help them change their understanding. In order to support the students' understanding, the teacher has to focus on how the students apprehend and discern phenomena related to the subject, rather than focusing on what they know about them or how they can manipulate them [7]. Consequently, with a teaching perspective viewing teaching as making understanding possible, the attention is directed toward the learning content, how it should be taught and how it is understood by the student. The teacher should focus on the essential issues that could represent critical barriers to student learning and give such issues special attention. Such teaching also involves discovering students' misunderstandings, intervening to change them and creating a context of learning that encourages students to engage with the subject matter [7]. For example, in supporting the students' use of theoretical knowledge in understanding the clinical situation, or to be able to discern and apprehend the most important information to learn from a clinical situation that might be useful in other situations. Although *Questions and answers *could be seen as a strategy facilitating and stimulating such a process with the student, we observed that this strategy was not applied in the same way by teachers. Some teachers provided more time for reflection and discussion, whilst others seemed to use this strategy more in order to assess students' knowledge content and level. This latter approach has previously been documented in the literature [8].

The strategies described in this study constituted the learning situation for the student. However, few statements indicated a deliberate use of the pedagogical strategies in order to facilitate learning by the clinical teacher. This is in accordance with other research describing clinical teachers' lack of arguments concerning how learning will best take place in clinical teaching [28]. Therefore, it is more likely that the strategies were learned traditionally and not deliberately used by the teacher.

### Pedagogical Implications of this study

This study may have pedagogical implications for clinical teaching in two different ways. Firstly, a greater knowledge of these pedagogical strategies, as well as meeting students and understanding their situational needs might assist clinical teachers in carrying out more effective teaching. Secondly, each of the described pedagogical strategies could be further explored to study how they could contribute to education and the enhancement of student learning.

## Competing interests

The authors declare that they have no competing interests.

## Authors' contributions

MSN, SP and EP have contributed to the design of the study. MSN and SP carried out the observations. MSN, SP and EP made the first preliminary analysis of data. MSN wrote the first draft of the paper. C-GW, EP and MSN contributed to the interpretation of the results and the final version of the manuscript. All authors read and approved the final manuscript.

## Authors' information

MSN is a doctoral student in Health Care Pedagogics.

SP has recently defended her thesis for a PhD exam in Health Care Pedagogics.

EP is Professor of Health Care Pedagogics with several years of experience of studies relating to clinical teaching and use of qualitative research methods, for example ethnographic methods.

C-GW is Professor of Education at University College of Kristianstad. He has a long experience of research on learning and teaching in Higher Education and from the application of qualitative research methods.

## Pre-publication history

The pre-publication history for this paper can be accessed here:

http://www.biomedcentral.com/1472-6920/10/9/prepub
